# Effect of Some Clinically Used Proteolytic Enzymes on Inflammation in Rats

**DOI:** 10.4103/0250-474X.40347

**Published:** 2008

**Authors:** A. H. M. Viswanatha Swamy, P A. Patil

**Affiliations:** 1 Department of Pharmacology and Pharmacotherapeutics, J. N. Medical College, Nehru Nagar, Belgaum - 580 010, India

**Keywords:** Inflammation, proteolytic enzymes, aspirin

## Abstract

The study was designed to investigate the role of three proteolytic enzymes *viz*., chymotrypsin, trypsin and serratiopeptidase on hind paw edema and cotton pellet induced granuloma and their possible interactions with aspirin in albino rats. Animals were treated with proteolytic enzymes alone in three different doses or aspirin or in combination with subantiinflammatory dose of aspirin or saline, 30 min before injection of 0.1ml 1% carrageenan. Paw volume was measured before and 3 h after the injection of carrageenan. Chymotrypsin, (5, 18 and 36 mg/kg), trypsin (1.44, 2.88 and 5.76 mg/kg) and serratiopeptidase (0.45, 0.9 and 2.70 mg/kg) were showed dose dependent antiinflammatory activity in acute model of inflammation. Serratiopeptidase showed better antiinflammatory activity on carrageenan induced inflammation than other two proteolytic enzymes and aspirin. However, chymotrypsin and serratiopeptidase were found to be more effective than aspirin in subacute model of inflammation. Chymotrypsin, trypsin and serratiopeptidase possess antiinflammatory activity and exhibit synergistic effect with aspirin in both acute and subacute models of inflammation in rats.

Inflammation is a normal response to protect the tissues from various noxious stimuli and is one of the most normal clinical conditions. A wide variety of enzymes and enzyme mixtures have been used as adjunctive therapeutic agents in a number of clinical conditions particularly in trauma and orthopedic clinics. Proteolytic enzymes are co-administered with non-steroidal antiinflammatory agents. Based on the earlier reports, it is suggested that the presence of proteolytic enzymes like chymotrypsin, cathepsin D^1^ and other proteases^2^ in inflammatory exudates indicate their role in the process of inflammation^3^–^5^. On the other hand, proteolytic activities of these enzymes have been proposed to be vital for the control of inflammation by clearing inflammatory debris^6^,^7^. There are several reports advocating the use of these proteolytic enzymes for the treatment of inflammatory disorders^8^,^9^. While other reports indicate that despite extensive clinical experience their antiinflammatory activity is unproved^5^ and controversial^8^. The literature survey indicates that there is paucity of information regarding serratiopeptidase and interaction with NSAID's. As controversies about the role of proteolytic enzymes like chymotrypsin, trypsin and serratiopeptidase on inflammation still exists, the present study was undertaken to probe antiinflammatory activity of these enzymes and their possible interactions with aspirin.

Wistar rats of either sex weighing between 120 and 150 g were used for the study. Animals were housed in a room temperature maintained at 22±1° with an alternating 12 h light-dark cycle. They were subjected to standard diet and water *ad libitum*. Trypsin, purchased from S. D. Fine Chemicals, Mumbai and aspirin was procured form Swastik Pharmaceuticals, Mumbai. Chymotrypsin and serratiopeptidase were purchased from the local market as alfapsin and bidanzen respectively. All the animal experimental protocol has been approved by the institutional animal ethics committee.

The clinical doses of all the three proteolytic enzymes used were converted to rat equivalents with the help of conversion Table^10^. All the drugs were dissolved in saline and were administered orally half an hour prior to the carrageenan injection and thereafter repeated once daily for 10 days in subacute model of inflammation.

Acute inflammation was produced by subplantar injection of 0.1 ml of 1% suspension of carrageenan in normal saline in the right hind paw of the rats^11^. Measurement of paw volume was done using plethysmograph at ‘0’ and ‘3’ h after injection of carrageenan as per the procedure given in the literature^12^. Oral administration of proteolytic enzymes (treatment/dose as shown in table) and aspirin is done 30 min before inducing inflammation and control group animals received normal saline. Mean increase in paw volume was measured and percentage inhibition was calculated by using the formula

% inhibition = 100(1-V_t_/V_c_)

where V_t_ is mean paw volume of drug treated, V_c_ mean paw volume of control.

Sub-acute inflammation was produced by cotton pellet induced granuloma in rats^13^,^14^. Sterile cotton (10±1 mg) soaked in 0.2 ml of distilled water containing penicillin (0.1 mg) and streptomycin (0.13 mg) was implanted subcutaneously bilaterally in axilla under ether anesthesia. The animals were treated with chymotrypsin (18 mg/kg), trypsin (2.88 mg/kg) and Serratiopeptidase (0.9 mg/kg) for consecutive 10 days. Saline treated animals served as control and aspirin (200 mg/kg) was administered as standard drug. The animals were sacrificed on the 11^th^ day and the cotton pellet granuloma was dissected out. After removal of fat and extraneous tissue, the cotton pellets were dried overnight at 55° and their dry weight was recorded. The weight of the cotton pellet before implantation was subtracted from the dried pellet to obtain the net granuloma weight.

Along with subacute antiinflammatory study, antiulcer activity was also performed by removing stomach and adrenal glands from animals treated with proteolytic enzymes, aspirin and control for studying gastric mucosa and weight changes in adrenal glands. The stomach thus removed were cut opened along the greater curvature and washed in saline. The severity of haemorrhagic erosions in the acid secreting glandular mucosa was assessed on a scale. The ulcer area was calculated under a dissecting microscope with a square grid. Gastric mucosal lesions were seen in the form of hemorrhages or linear breaks. Ulcer index was calculated using the following method^15^. Ulcer index = 10/X, where X = Total mucosal area/total area of mucosal lesions. The results were analyzed by Student's t test and *P* values <0.05 were considered to be significant.

In acute inflammation model, the carrageenan induced paw edema was significantly reduced by the proteolytic enzymes when compared to control (Table 1). The percentage protection (inhibition) of edema for chymotrypsin (36 mg/kg), trypsin (5.76 mg/kg) and serratiopeptidase (2.70 mg/kg) and aspirin (200 mg/kg) was found to be 62.03, 49.07, 62.81 and 56.09, respectively. The lowest dose of all the three enzymes and aspirin (54 mg/kg) individually has failed to show antiinflammatory activity, but in combination have shown antiinflammatory activity. In the model of subacute inflammation, % weight of the granulation tissue was significantly reduced in animals treated with chymotrypsin (42%), trypsin (36%) and serratiopeptidase (45%) and its combination with subantiinflammatory dose of aspirin showed antiinflammatory effect (Table 2). The chymotrypsin, trypsin and serratiopeptidase treated animals showed significant reduction in ulcer index as compared to control and its combination with aspirin as compared to aspirin treated animals (Table 3).

**TABLE 1:** EFFECT OF PROTEOLYTIC ENZYMES AND STANDARD DRUG ON CARRAGEENAN INDUCED PAW EDEMA

Treatment	Dose (mg/kg)	Edema at 3^rd^ h	% Inhibition
Control	Normal saline	1.08 ± 0.03	
Aspirin	200.00	0.18 ± 0.03	56.09*
Chymotrypsin	9.00	0.98 ± 0.24	9.25
Chymotrypsin	18.00	0.73 ± 0.12	32.40*
Chymotrypsin	36.00	0.41 ± 0.06	62.03*
Trypsin	1.44	0.83 ± 0.11	23.14
Trypsin	2.88	0.70 ± 0.10	35.18*
Trypsin	5.76	0.55 ± 0.05	49.07*
Serratiopeptidase	0.45	1.03 ± 0.04	4.62
Serratiopeptidase	0.90	0.47 ± 0.18	56.48*
Serratiopeptidase	2.70	0.38 ± 0.05	62.81*
Chymotrypsin + Aspirin	9.00 + 54.00	0.43 ± 0.04	60.18*
Trypsin + Aspirin	1.44 + 54.00	0.61 ± 0.04	43.51*
Serratiopeptidase + Aspirin	0.45 + 54.00	0.16 ± 0.02	60.97*

Values are mean ± SEM; *n* = 6 animals in each group;

* *p* < 0.05 when compared to control

**TABLE 2:** EFFECT OF PROTEOLYTIC ENZYMES AND STANDARD DRUG ON COTTON PELLET INDUCED GRANULOMA FORMATION

Treatment	Dose (mg/kg)	Mean granuloma wt (mg/100 g)	% Inhibition
Control	Normal Saline	45.54 ± 1.19	
Aspirin	200.00	27.66 ± 2.60	39.24*
Chymotrypsin	18.00	26.46 ± 2.88	41.88*
Trypsin	2.88	29.22 ± 2.43	35.85*
Serratiopeptidase	0.90	25.15 ± 1.13	44.77*
Chymotrypsin + Aspirin	9.00 + 54.00	28.88 ± 1.82	36.58*
Trypsin + Aspirin	1.44 + 54.00	30.14 ± 0.58	33.00*
Serratiopeptidase + Aspirin	0.45 + 54.00	28.10 ± 4.23	38.28*

Values are mean ± SEM; *n* = 6 animals in each group;

* *p* < 0.05 when compared to control

**TABLE 3:** EFFECT OF PROTEOLYTIC ENZYMES AND STANDARD DRUG ON GASTRIC MUCOSA (ULCER INDEX)

Treatment	Dose (mg/kg)	Mean ulcer index ± SEM	% Inhibition
Control	Normal Saline	10.00 ± 6.32	
Aspirin	200.00	40.00 ± 0.00	
Chymotrypsin	18.00	5.00 ± 0.60	50.00*
Trypsin	2.88	5.60 ± 0.66	44.00*
Serratiopeptidase	0.90	4.21 ± 0.26	57.90*
Chymotrypsin + Aspirin	9.00 + 54.00	26.42 ± 5.83	33.95#
Trypsin + Aspirin	1.44 + 54.00	30.00 ± 4.21	25.00#
Serratiopeptidase + Aspirin	0.45 + 54.00	23.33 ± 7.602	41.67#

Values are mean ± SEM; *n* = 6 animals in each group;

* *p* < 0.05 when compared to control and

# *p* < 0.05 when compared to aspirin

The proteolytic enzymes treated animals showed significant increase in adrenal gland weights as compared to control, i.e., chymotrypsin showed 71.71% increase in adrenal gland weight while trypsin and serratiopeptidase showed 39.97% and 89.94%, respectively. But in aspirin treated group the adrenal gland weight was decreased significantly as compared to control (Table 4).

**TABLE 4:** EFFECT OF PROTEOLYTIC ENZYMES AND STANDARD DRUG ON WEIGHT OF ADRENAL GLANDS

Treatment	Dose (mg/kg)	Mean weight (mg%BW)	% Increases
Control	Normal saline	9.58 ± 0.767	
Aspirin	200.00	5.38 ± 0.23	43.84.†*
Chymotrypsin	18.00	16.45 ± 1.81	71.71*
Trypsin	2.88	13.41 ± 1.24	39.97*
Serratiopeptidase	0.90	18.12 ± 1.93	89.94
Chymotrypsin + Aspirin	9.00 + 54.00	13.25 ± 1.02	38.30*
Trypsin + Aspirin	1.44 + 54.00	15.54 ± 2.01	62.21*
Serratiopeptidase + Aspirin	0.45 + 54.00	17.83 ± 3.07	86.11*

Values are mean ± SEM; *n* = 6 animals in each group;

* *p* < 0.05 when compared to control.

† : % decrease

Findings of the present study clearly indicate that chymotrypsin, trypsin and serratiopeptidase have suppressed inflammation significantly both in carrageenan as well as cotton pellet induced granuloma and appear to be dose dependent. The lowest dose of all the three enzymes and aspirin individually has failed to show any significant antiinflammatory activity on carrageenan induced inflammation, but in combination of all the above have pontentiated antiinflammatory activity of aspirin. The potentiated antiinflammatory activity of aspirin was comparable to that of aspirin 200 mg/kg, not only in carrageenan induced but also in cotton pellet induced granuloma study. The antiinflammatory activity of these enzymes in both models of inflammation may be attributed due to stimulation of neutrophil apoptosis^16^, inhibition of neutrophil migration at the inflammatory site^17^, inhibition of bradykinin synthesis, decreased vascular permeability and by clearing inflammatory debris^18^–^20^.

Aspirin has expected to produce significant increase in the ulcer index as compared to control. While chymotrypsin, trypsin and serratiopeptidase alone and in combination with aspirin reduced ulcer index significantly as compared to aspirin treated animals. The reduction in ulcer index observed in this study may be due to boost of the defensive factors.

Adrenal gland weights of the animals treated with all the three proteolytic enzymes were significantly increased as compared to control but in contrast to aspirin treated animals in which it decreased significantly as compared to control. The reduction in the weight of adrenal glands in aspirin treated group may be because of production of corticosteroids which may in turn explain the antiinflammatory activity of aspirin^21^. Increase in adrenal glands weight in proteolytic enzymes treated groups may indicate the release of catecholamines that are responsible for antiinflammatory activity^22^,^23^. Further investigations are needed to establish these facts by biochemical estimations of corticosteroids and catecholamines in plasma.

The observation of the present study clearly indicates that antiinflammatory activity of all the three enzymes were dose dependent. The combination of low doses of these enzymes with sub antiinflammatory dose of aspirin resulted in synergistic antiinflammatory activity without ulcerogenic potential appears to be clinically a beneficial interaction. If the present findings could be extrapolated to human beings, such combination therapies may reduce the adverse effects of NSAID's like aspirin. However clinical trials are further needed to be studied to confirm the same.
